# Immunotherapy in non-small cell lung cancer: rationale, recent advances and future perspectives

**DOI:** 10.1093/pcmedi/pbab027

**Published:** 2021-12-02

**Authors:** Wenxin Luo, Zhoufeng Wang, Ting Zhang, Lan Yang, Jinghong Xian, Yalun Li, Weimin Li

**Affiliations:** Department of Respiratory and Critical Care Medicine, West China Hospital, Sichuan University, Chengdu 610041, China; Precision Medicine Research Center, West China Hospital, Sichuan University, Chengdu 610041, China; Clinical Medical College and the First Affiliated Hospital of Chengdu Medical College, Chengdu 610500, China; Department of Respiratory and Critical Care Medicine, West China Hospital, Sichuan University, Chengdu 610041, China; Department of Clinical Research Management, West China Hospital, Sichuan University, Chengdu 610041, China; Department of Respiratory and Critical Care Medicine, West China Hospital, Sichuan University, Chengdu 610041, China; Department of Respiratory and Critical Care Medicine, West China Hospital, Sichuan University, Chengdu 610041, China; Precision Medicine Research Center, West China Hospital, Sichuan University, Chengdu 610041, China

**Keywords:** non-small cell lung cancer, immunotherapy, immune checkpoint inhibitors, adoptive cell therapy, vaccine

## Abstract

Lung cancer, with non-small cell lung cancer (NSCLC) being the major type, is the second most common malignancy and the leading cause of cancer-related death globally. Immunotherapy, represented by immune checkpoint inhibitors (ICIs), has been one of the greatest advances in recent years for the treatment of solid tumors including NSCLC. However, not all NSCLC patients experience an effective response to immunotherapy with the established selection criteria of programmed death ligand 1 (PD-L1) and tumor mutational burden (TMB). Furthermore, a considerable proportion of patients experience unconventional responses, including pseudoprogression or hyperprogressive disease (HPD), immune-related toxicities, and primary or acquired resistance during the immunotherapy process. To better understand the immune response in NSCLC and provide reference for clinical decision-making, we herein review the rationale and recent advances in using immunotherapy to treat NSCLC. Moreover, we discuss the current challenges and future strategies of this approach to improve its efficacy and safety in treating NSCLC.

## Introduction

Lung cancer, with non-small cell lung cancer (NSCLC) being the major type, is the second most common malignancy and the leading cause of cancer-related death worldwide. According to the latest Global Cancer Statistics 2020, the estimated number of new lung cancer cases in the world was 2.206 million in 2020, accounting for 11.4% of all new malignancies; and the estimated number of lung cancer deaths in the world was 1.796 million in 2020, accounting for 18.0% of all cancer deaths.^1^ At present, the treatment of NSCLC mainly includes surgery, chemotherapy, radiotherapy, molecular targeted therapy and immunotherapy, depending on the specific stage and condition. Since the majority of patients with lung cancer are diagnosed at an advanced stage, they usually have missed the opportunity of radical surgery treatment.^2^ For advanced NSCLC, chemotherapy has long been the major treatment, but it seems to have limited effect. Molecular targeted therapy such as EGFR-TKI and ALK-TKI have become the standard first-line therapy for patients with advanced NSCLC with positive driver gene mutations, significantly prolonging the survival period and improving the quality of life of patients. However, the “bottleneck” of molecular targeted therapy lies in that secondary mutations often occur during treatment, resulting in drug resistance.^3,4^ After chemotherapy and molecular targeted therapy, the treatment of advanced NSCLC has entered a new era of immunotherapy represented by immune checkpoint inhibitors (ICIs).^5^

Immunotherapy is a relatively new treatment approach for cancers including NSCLC, which is hoped to further improve the prognosis of NSCLC. This review discusses our current knowledge of the immune response in NSCLC, the latest and ongoing immune-based therapies, and the future of immunotherapies in NSCLC.

## Tumor immunology and immunotherapy in NSCLC

### The wrestling between cancer and immunity

**Figure 1.**
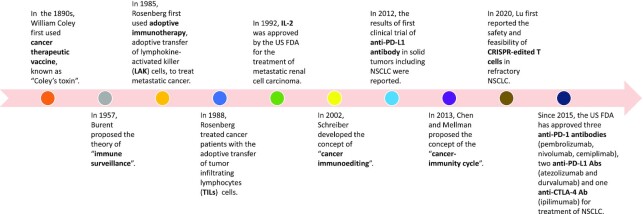
History of cancer immunotherapy.

Under normal physiological conditions, the “surveillance function” of human immune system enables it to identify and eliminate foreign components, including invading pathogenic microorganism, allografts, and tumor cells. Though such function of recognizing and killing tumors was first proposed as a hypothesis in as early as 1909,^6^ it was not until 50 years later that Prehn and Main demonstrated the presence of specific antigens in tumor cells and adaptive immune response of host in experimental animal model.^7^ In 1957, Burent proposed the theory of “immune surveillance”, holding that nascent transformed cells may arise in our bodies and the immune system can recognize and eradicate these transformed cells before they are clinically manifested.^8^ In 2002, Schreiber *et al*. developed the concept of “cancer immunoediting” that there are complex interactions between tumor and immune system in the process of tumor development, mainly consisting of three phases: (1) elimination phase, in which the immune system effectively recognizes and attacks early tumor;  (2) equilibrium phase, where the killing of tumor by immune system and the growth of tumor are in a dynamic equilibrium state; and (3) escape stage, in which tumor grows and metastasizes by escaping from the recognition and eradication of immune system through different mechanisms (Fig. 1).^9^

**Figure 2.**
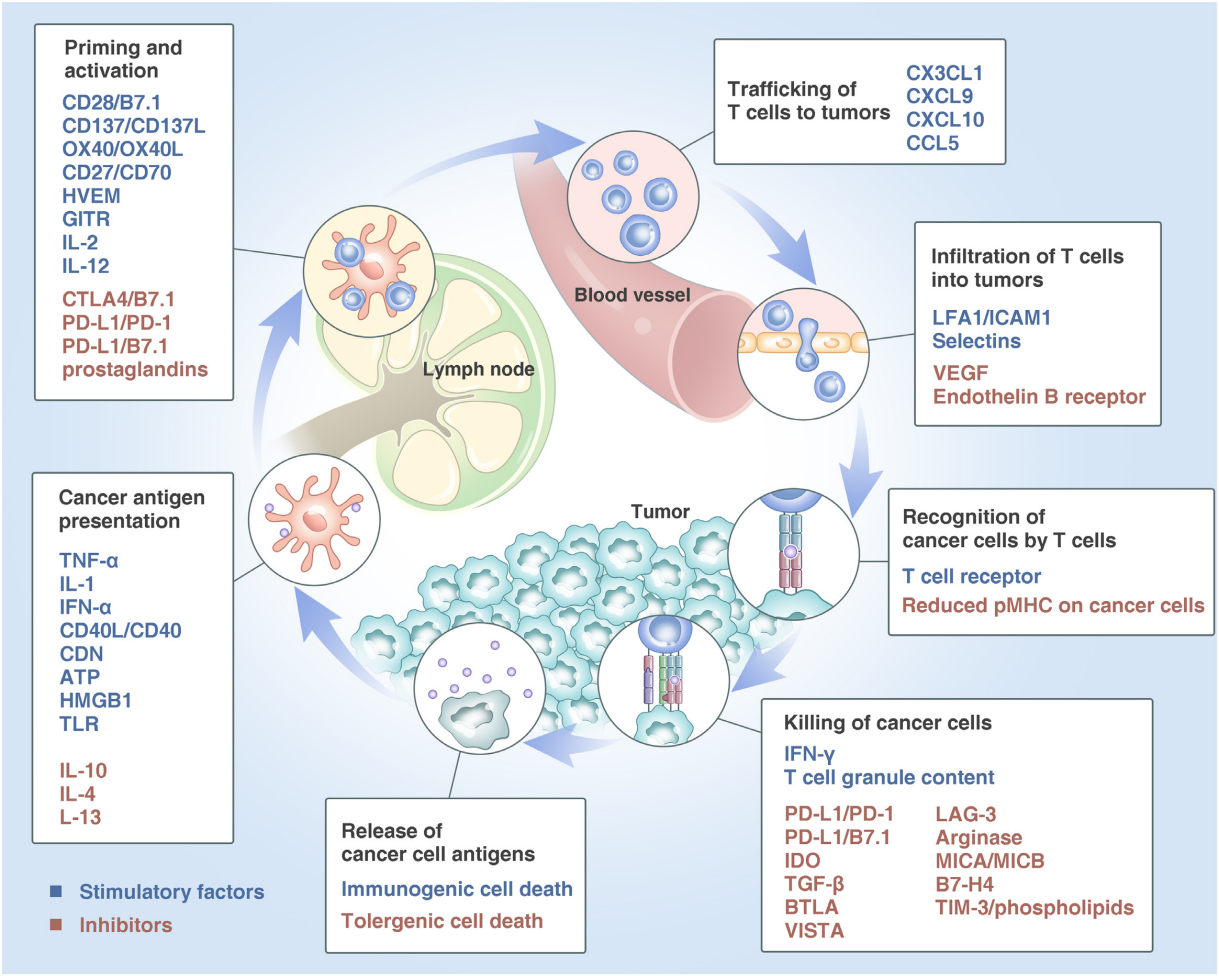
The “cancer-immunity cycle”. Figure adapted from Ref. 10. Copyright Elsevier, 2013.

As our understanding of the relationship between tumor and immunity deepens, we now know that for immune system's effective killing of cancer cells, a series of stepwise events must be initiated and allowed to proceed and expand iteratively. The “cancer-immunity cycle” proposed by Chen and Mellman in 2013 reveals the mechanism by which the immune system recognizes and eradicates tumor cells. The cancer-immunity cycle is divided into seven steps (Fig. 2).^10,11^ (1) Release of cancer cell antigen. Cancer cell antigens, created by cancer cell deaths, genetic alterations and cancer differentiation etc, lead to expression and binding of antigen peptides to major histocompatibility class (MHC) molecules on the surface of cancer cells, distinguishing them from their normal counterparts. (2) Cancer antigen presentation. Antigen presenting cells (APCs), mainly dendritic cells (DCs), capture cancer-specific antigen peptide by binding antigen peptide to MHC molecules on the surface of APCs, and subsequently present them to T cells. (3) Priming and activation. In lymph node, T cell receptor (TCR) recognizing the antigen/MHC complex on the APC surface, as well as the interaction between CD28 molecule on the T cell surface and the B7.1 molecule on the APC surface, prime and activate the T cells. (4) Trafficking of T cells to tumor. The activated effector T cells traffic to the tumor bed through blood circulation. (5) Infiltration of T cells into tumors. Effector T cells migrate from the circulating blood to the tumor bed across the vascular endothelial barrier. (6) Recognition of cancer cells by T cells. Cytotoxic T lymphocytes (CTLs) specifically recognize and bind to cancer cells through the interaction between its TCR and antigen/MHC complex on the cancer cells. (7) Killing of cancer cells. CTLs kill their target cancer cells, lead to releasing additional cancer antigens (step one) and subsequent another circulation of the cycle. Through the mechanism of cancer-immunity cycle above, the host immune system can effectively kill cancer cells.

However, such cancer-immunity cycle does not always perform perfectly in cancer patients. Tumors can escape from the host immune system through changing tumor cells themselves or the tumor microenvironment (TME), thus maintaining the continuous proliferation and invasion of tumor cells and eventually leading to the occurrence and development of tumor, which is called “immune escape”.^12^ The immune escape of tumor is essentially achieved by disrupting certain steps in the cancer-immunity cycle.

### Rationale for immunotherapy against NSCLC

At each step of the cancer-immunity cycle, there are positive and negative regulators that keep the activation of the immune system within the normal range.^10^ Therefore, we can achieve the therapeutic purpose through strengthening the positive regulation signals or suppressing the negative regulation signals.

Directing at the negative immune checkpoints signaling, ICIs are the most developed and widely-used strategy against NSCLC.^13^ Immune checkpoint is a class of immunosuppressive molecules, which are expressed on immune cells and can regulate the degree of immune activation. Cytotoxic T lymphocyte associated antigen-4 (CTLA-4) and programmed cell death protein-1 (PD-1) are the representatives of immune checkpoints in NSCLC, which act as inhibitors in the activation of T cells.^14^ CTLA-4 is presented on T cells and its interaction with B7 on APCs reduces IL-2 production and T cell proliferation in lymphoid organs. Such reduction can be blocked by CTLA-4 inhibitors.^15^ Besides, several recent studies found that CTLA-4 inhibitors exhibit antitumor function by selectively depleting regulatory T (Treg) cells in the TME through an Fc-dependent mechanism.^16–18^ PD-1 is expressed on activated T cells, and its ligand programmed cell death protein ligand-1 (PD-L1) can be expressed on the surface of tumor cells and immune cells. The binding of PD-1 and PD-L1 can inhibit activated T cell proliferation, promote activated T cell apoptosis and reduce cytokine secretion in the TME.^15^ Apart from CTLA-4 and PD-1, novel immune checkpoint molecules on T cells have been discovered, including TIGIT, LAG-3, TIM-3, VISTA and CD244.^19–23^ These immune checkpoints can inhibit T cell function by binding to their ligands on tumor cells, APC cell or other cells. For instance, TIGIT is an immune checkpoint mainly expressed on the surface of T cells and natural killer (NK) cells and can inhibit cell function by binding to its ligands CD55 and CD122; LAG-3 is expressed on activated CD4+, CD8+ T cells and NK cells, and can inhibit T cell function by binding to its ligand FGL1. Blocking these immune checkpoints can achieve reactivation of T cells and NK cells to enhance the antitumor activity.

Adoptive cell therapy (ACT) and cancer vaccine are other two promising immunotherapy strategies for patients with NSCLC. ACT, including chimeric antigen receptor (CAR) T-cell therapy, engineered T-cell receptor (TCR) T-cell therapy, tumor-infiltrating lymphocyte (TIL) therapy and so on, aimed at reprograming immune cells to enhance tumor cells’ recognizing and killing.^24,25^ Cancer vaccines, including tumor antigen associated vaccines, neoantigen associated vaccines and cell vaccines, are designed to amplify tumor-specific T cell responses via active immunization.^26,27^

### Recent advance of immunotherapy in NSCLC

In the past decade, ICIs treatment has achieved significant progress in NSCLC.^15,28,29^ First, the use of ICIs has expanded from the initially second-line therapy to multiple clinical settings, including neoadjuvant, adjuvant, first, second, and subsequent lines treatment. Second, ICIs treatment has expanded from monotherapy to combination therapy, including combination of different types of ICIs (i.e. PD-1/PD-L1 inhibitors with CTLA-4 inhibitors), as well as ICI treatment with chemotherapy, radiology, and chemotherapy plus anti-VEGF antibody. Third, several novel ICIs targeting LAG-3, TIM-3 and VISTA are undergoing clinical trials to evaluate their efficacy and safety in treatment of solid tumors including NSCLC and exhibit great therapeutic promise.^30–33^ Currently, three anti-PD-1 monoclonal antibodies (mAbs; pembrolizumab, nivolumab, cemiplimab), two anti-PD-L1 mAbs (atezolizumab and durvalumab) and one anti-CTLA-4 mAb (ipilimumab) have been approved by the US Food and Drug Administration (FDA) for treatment of NSCLC.^34,35^ Besides, the China National Medical Products Administration (NMPA) has approved four more mAbs that target PD-1 (camrelizumab, sintilimab, tiselizumab and toripalimab) for treatment of NSCLC.^36^ More recently, novel anti-TIGIT mAb tiragolumab is approved by FDA for treatment of NSCLC (Table 1).

**Table 1.** Currently approved immunotherapy in NSCLC.

Type	Regimen	FDA approval	NMDA approval
PD-1 mAb	Pembrolizumab	First and second line treatment for squamous/non-squamous advanced NSCLC	First line treatment for advanced squamous/non-squamous NSCLC
PD-1 mAb	Nivolumab	Second line treatment for squamous/non-squamous advanced NSCLC	Second line treatment for squamous/non-squamous advanced NSCLC
PD-1 mAb	Cemiplimab	First line treatment	/
PD-L1 mAb	Atezolizumab	First and second line treatment for squamous/non-squamous advanced NSCLC	/
PD-L1 mAb	Durvalumab	Unresectable stage III NSCLC	Unresectable stage III NSCLC
PD-1 mAb	Camrelizumab	/	First line treatment for advanced non-squamous NSCLC
PD-1 mAb	Sintilimab	/	First line treatment for advanced non-squamous NSCLC
PD-1 mAb	Tiselizumab	/	First line treatment for squamous advanced NSCLC
PD-1 mAb	Toripalimab	/	First line treatment for advanced NSCLC
CTLA4 mAb	Ipilimumab	First line treatment	First line treatment
TIGIT mAb	Tiragolumab	First line treatment	/

### ICIs monotherapy as first-line treatment for advanced NSCLC

Five phase III trials reported outcomes for first-line ICIs monotherapy in advanced NSCLC. In KEYNOTE-024, KEYNOTE-042, Impower 110, pembrolizumab and atezolizumab showed significantly improved overall survival (OS) compared with chemotherapy in patients with advanced NSCLC.^37–42^ On the other hand, CheckMate 026 trial reported that nivolumab failed to prolong progression-free survival (PFS) and OS when compared with chemotherapy, indicating not all ICIs monotherapies as first-line therapy can be helpful for advanced NSCLC.^43^ More recently, EMPOWER-Lung 1 trial reported its efficacy and safety outcomes of cemiplimab monotherapy. Significant improvements in OS (median: not evaluable versus 14.2 months) and PFS (median: 8.2 months versus 5.7 months) were observed in patients who received cemiplimab monontherapy compared to those underwent chemotherapy. Moreover, lower frequency of grade 3–4 immune-related adverse events (irAEs) occurred in patients treated with cemiplimab than in those treated with chemotherapy (28% versus 39%).^44,45^ Based on results of these trials, pembrolizumab, atezolizumab and cemiplimab monotherapy have been approved for first-line therapy in advanced NSCLC.

### ICIs-based combination therapy as the first-line treatment for advanced NSCLC

Multiple completed phase III trials have evaluated the efficacy and safety of ICIs-based combination therapy (including PD-1/PD-L1 inhibitors plus chemotherapy, PD-1/PD-L1 inhibitors plus chemotherapy plus anti-angiogenetic therapy, PD-1/PD-L1 inhibitors plus CTLA-4 inhibitors, and PD-1/PD-L1 inhibitors plus CTLA-4 inhibitors plus chemotherapy) as the first-line treatment in advanced NSCLC. In these trials, pembrolizumab-chemotherapy (KEYNOTE-021, KEYNOTE-189 and KEYNOTE-407),^46–49^ atezolizumab-chemotherapy (IMpower 130),^50^ atezolizumab-bevacizumab-chemotherapy (IMpower 150),^51^^52^ nivolumab-ipilimumab (CheckMate 227)^53^ and nivolumab-ipilimumab-chemotherapy (CheckMate 9LA)^54^ showed significantly improved OS, PFS and objective response rate (ORR) compared with controls in patients with advanced NSCLC; and subsequently are approved for first-line treatment of advanced NSCLC. Notably, FDA approved another ICI tiragolumab in 2021, based on the results of phase II CITYSCAPE trial, demonstrating that combination of TIGIT and PD-L1 inhibitors may enhance antitumor activity by potentially amplifying the immune response. In the CITYSCAPE trial, comparable PFS improvement with tiragolumab plus atezolizumab relative to atezolizumab monotherapy was seen in PD-L1–high NSCLC patients (PFS hazard ratio (HR) 0.23, 95% CI: 0.10–0.53).^55^

### ICIs monotherapy as second-line treatment for advanced NSCLC

ICIs monotherapy pembrolizumab, nivolumab and atezolizumab have been approved by FDA/NMPA for the second-line treatment in advanced NSCLC based on improved survival and safety data from five phase III clinical trials (KEYNOTE-010, OAK, CheckMate 078, CheckMate 017 and CheckMate 057).^56–61^

### ICIs neoadjuvant therapy for early-stage resectable NSCLC

Although ICIs have yet been approved for the neoadjuvant treatment in NSCLC, reported efficacy data from trials has been promising and ICIs will likely play an important role in the treatment of early-stage resectable NSCLC. In the setting of neoadjuvant monotherapy with ICIs, series of trials have demonstrated that ICIs (nivolumab, atezolizumab and sintilimab) have great potentials with higher major pathologic response (MPR) and pathological complete response (pCR) when compared with chemotherapy.^62–64^ For ICIs-based neoadjuvant combination therapy, two recently completed studies have reported efficacy outcomes in patients treated with neoadjuvant chemoimmunotherapy. In the phase II trial of toripalimab plus chemotherapy as neoadjuvant treatment in resectable stage III NSCLC (NeoTPD01 Study) with 30 out of the total 33 enrolled patients undergoing resection, the MPR rate was 66.7% (20/30), the pCR rate was 50% (15/30), and 96.7% (29/30) patients achieved R0 resection.^65^ The phase III Checkmate-816 trial, which aimed to evaluate nivolumab plus chemotherapy versus chemotherapy as neoadjuvant therapy for resectable stage IB-IIIA NSCLC, published its latest results at the 2021 AACR congress. NIVO plus chemo significantly improved pCR compared to chemo (24.0% versus 2.2%, P < 0.0001), MPR (36.9% versus 8.9%), as well as ORR (53.6% versus 37.4%). Furthermore, neoadjuvant treatment did not cause death or delay in surgery.^66^

### ICIs adjuvant therapy for early-stage resectable NSCLC

Similar to neoadjuvant immunotherapy, safety and efficacy of adjuvant immunotherapy in patients with early-stage resectable NSCLC are being explored in multiple phase II to III trials. Recently, the primary results were released for the phase III IMpower010 trial, which assessed the safety and efficacy of atezolizumab versus best supportive care (BSC) after adjuvant chemotherapy in resected stage IB-IIIA NSCLC. Atezolizumab showed statistically significant disease-free survival (DFS) benefit versus BSC (36 months: 60.0% versus 48.2%).^67^ Two main trials of adjuvant therapy with anti-PD-1 agents, ANVIL (nivolumab) and PEARLS (pembrolizumab), are underway and efficacy outcomes have yet to be published.^68^

### ICIs consolidation therapy for unresectable stage III NSCLC

PACIFIC was a phase III trial in patients with unrectable stage III NSCLC treated with consolidative durvalumab or placebo after concurrent chemoradiotherapy. Median PFS were 16.8 months in the duvalizumab group versus 5.6 months in the placebo group (HR = 0.51; 95%CI: 0.41–0.63).^69^ Based on this result, FDA and NMDA approved duvalizumab as consolidation therapy in unresectable stage III NSCLC. In 2021, PACIFIC reported its latest efficacy outcomes. Estimated 4-year OS rates were 49.6% for durvalumab versus 36.3% for placebo, and 4-year PFS rates were 35.3% (duravlumab) versus 19.5% (placebo).^70^

### Adoptive cell therapy (ACT) and cancer vaccine for advanced NSCLC

So far, TCR-T has gone through four iterations.  In recent years, TCR-T therapy worldwide has mainly targeted solid tumors including NSCLC. For instance, ADP-A2M4CD8, a novel TCR-T therapy, which coexpress the CD8 coreceptor with the engineered TCR targeting MAGE-A4, is currently being investigated for the treatment of solid tumors.  In the ongoing phase 1 SURPASS trial, which enrolled multiple solid tumors including NSCLC, the majority of evaluable patients (13/15) had evidence of disease control and there were RECIST responses in several types of solid tumor.^71^ On the other hand, scientists developed several cancer vaccines, L-BLP25, MAGE-A3, TG4010, NY-ESO-1, CIMAvax-EGF and others in the past two decades.^72–75^ Among them, CIMAvax-EGF, which is built on the induction of a specific immune response, aiming to sequester EGF, showed ideal efficiency in clinical trials. A phase III trial enrolled stage IIIB/IV NSCLC patients and randomly assigned to receive CIMAvax-EGF or placebo, and found significantly increased median survival time in patients in CIMAvax-EGF group, and CIMAvax-EGF was well tolerated.^76^ More recently, another vaccine OSE2101, which modifies epitopes restricted to HLA-A2+ from five tumor-associated antigens, was demonstrated to have better prognosis (median OS: 11.1 months vs 7.5 months, HR 0.59) and fewer severe adverse events (38% vs 68%, p < 0.001) compared with standard of care in advanced NSCLC.^77^ Currently, multiple clinical trials assessing ACT and cancer vaccine in NSCLC are still underway.

## Challenges and perspectives of immunotherapy in NSCLC

### Screening of potential benefit population

Numerous clinical trials and studies have confirmed that only a small fraction of NSCLC patients show objective responses to immunotherapy and get long-term benefit from immunotherapy; nevertheless, there are currently no optimal predictors to identify patients who will likely benefit from immunotherapy.

#### PD-L1, TMB and dMMR/MSI-H

At present, biomarkers including PD⁃L1, tumor mutational burden (TMB) and mismatch repair deficient (dMMR)/microsatellite instability-high (MSI-H) have shown some predictive value, and are approved by the FDA and/or the NMPA as indicators for predicting the efficacy of immunotherapy in NSCLC or other solid tumors. Among these markers, PD-L1 is the most widely-used in NSCLC. However, even for those advanced NSCLC patients with relatively high PD-L1 expression (≥50% of tumor cells or ≥ 10% of tumor-infiltrating immune cells), NSCLC patients receiving first-line monotherapy with PD-1/PD-L1 inhibitors (pembrolizumab and atezolizumab) had ORR of about 38.3%–46.1%.^37,39,41,42^ For TMB, the ORR in NSCLC patients with TMB high (TMB-H, defined as TMB ≥ 10 mutations/Mb) treated with nivolumab plus ipilimumab was only around 33%-48%, according to the previous trials.^78–80^ Although recent studies suggested that dMMR/MSI-H may be a predictor for PD-1 inhibitors therapy regardless of the cancer origin, the incidence of dMMR/MSI-H in NSCLC is very low and their predictive value for NSCLC needs further verification through more clinical trials and studies.^81–83^ To improve forecasting ability, scholars analyzed the predictive utility of combination of PD-L1 expression and TMB in NSCLC, and found that combined use of PD-L1 expression and TMB is a promising biomarker to evaluate patients’ survival after immunotherapy (1-year PFS AUC 0.826; 3-year PFS AUC 0.948).^84^

#### Predictive model

Studies have shown that the small fraction of patients who get improved clinical outcomes tend to have some common features, including male, smoking history, and good general physical condition, with performance status (PS) score of 0–1.^85^ Thus, predictive models were built based on these features to help screening potential target population. So far the best predictive model for NSCLC immunotherapy is iSEND, which includes gender, PS score, Neutrophil-to-lymphocyte ratio (NLR), and Delta NLR as the variables to categorize patients into different risk groups and significantly discriminates each group's clinical outcome.^86,87^ Although these data are from small-scale clinical studies, predictive models based on integrative analysis of real-world data should be promising in screening potential benefit populations in today's environment of big data analysis and artificial intelligence.

#### ctDNA

Detection of circulating tumor DNA (ctDNA) has not only value in precise diagnosis of NSCLC (i.e. driver mutations, TMB and MMR), but also probable potential in predicting the efficacy of immunotherapy in NSCLC.^88–90^ For instance, Sarah *et al*. showed that in metastatic NSCLC receiving ICIs, a ctDNA response (defined as a > 50% decrease in mutant allele fraction from baseline) was associated with superior PFS (HR = 0.29, P = 0.03), and superior OS (HR = 0.17, P = 0.007); besides, the decline in ctDNA levels preceded the imaging confirmation of tumor shrinkage (24.5 days versus 72.5 days).^91^ Thus, monitoring ctDNA levels in NSCLC patients receiving ICIs enables early assessment of immunotherapy response and might avoid the prolonged administration of ineffective treatments.

#### Immune-related toxicity

Immunotherapy has significantly prolonged the patient survival, but it also brings immune-related toxicity, or irAEs. Notably, more and more studies have suggested that development of irAEs predicts a better response to immunotherapy in NSCLC.^92–96^ In 2018, scholars first reported that the PFS and OS of patients with irAEs were significantly better than those without adverse reactions after treatment with nivolumab (*P* = 0.04 and 0.01, respectively); the ORR of patients with irAEs was significantly higher than that of patients without irAEs (52.3% and 27.9%, respectively).^93^ Further studies found that among the various types of irAEs, endocrine toxicity and dermatological toxicity may be most closely related to the efficacy of immunotherapy.^92^ However, the mechanism remains not clear at present, though it is speculated to be related to the important role of immune checkpoint in maintaining the process of autoimmune balance.

Apart from the predictors mentioned above, CD8+ T-cell tumor-infiltrating, genetic mutations (RYR1, MGAM and STK11), copy number alteration and HLA class I diversity are also being explored for treating NSCLC and other solid tumors.^84,97–99^ Taken together, there cannot be a single perfect predictor to screen potential target populations for immunotherapy in NSCLC; predictive models are needed that take into account different parameters affecting tumor-host interactions.

### Objective evaluation of response to immunotherapy

Different from chemotherapy, radiotherapy, or molecular targeted therapy, ICIs do not exert direct cytotoxic effects on tumor cells, but restore or enhance the immune system's antitumor response with immune cells as the target. The complexity of the response patterns after immunotherapy warrants special attention.

At present, four unconventional response patterns for immunotherapy have been observed: pseudoprogression, delayed response, mixed response, and hyperprogressive disease (HPD).^100,101^ Pseudoprogression is an initial increase in the tumor volume or number of tumor lesions followed by a decrease.^102^ The reported incidence of pseudoprogression is 2.6%–4.7% in NSCLC.^102–105^ In 2017, the RECIST Working Group officially proposed a modified RECIST 1.1 for immune-based therapeutics (termed iRECIST).^106^ The iRECIST criteria introduce the concepts of immune unconfirmed progressive disease (iUPD) and immune confirmed progressive disease (iCPD). A progressive disease (PD) previously assessed by traditional RECIST 1.1 is temporarily evaluated as iUPD, and continuation of treatment is determined based on the tumor type, disease stage and clinical situation of the patient; it can only be confirmed as iCPD by re-evaluation at 4–8 weeks. This reassessment approach can identify unconventional responses such as pseudoprogression and delayed responses. Moreover, serological biomarkers, including ctDNA and NLR, might help distinguish pseudoprogression from true progression.^107–109^

HPD is featured with drastic progression of disease after immunotherapy, but there has been no standard definition. Kato *et al*. first defined HPD with three criteria: time to treatment failure (TTF) <2 months, > 50% increase in tumor burden and > 2-fold increase in progression rate.^110^ Russo *et al*. later required three out of five criteria for being diagnosed with HPD, including TTF < 2 months, ≥ 50% increase in the sum of the diameter of target lesions, appearance of at least two new lesions in an affected organ, dissemination to a new organ or clinical deterioration to PS ≥ 2.^111^ The reported incidence of HPD ranged from 9.2% to 17.9% in NSCLC due to application of different criteria.^105,112,113^ The prognosis of patients with HPD is extremely poor with median OS of 1.7–3.4 months.^105,114,115^ Once HPD is suspected, immunotherapy should be interrupted and a detailed evaluation should be conducted immediately. However, there are currently no reliable predictors for HPD after immunotherapy. Thus, the recognition of HPD warrants further studies.

### Management of immune-related toxicity

The immune-related toxicity induced by ICIs not only limits the use of these beneficial drugs, but also threatens the patient's health. The irAEs can occur in all tissues and organs throughout the body, including skin, endocrine system, lung, liver, gastrointestinal system, musculoskeletal system, nervous system, cardiovascular system, eyes, hematologic system, and others.^116–119^ Although the overall incidence of irAEs is low in NSCLC, some of them can be severe and even life-threatening, requiring early accurate recognition and adequate management.

Searching for predictive biomarkers of irAEs, which is crucial for early diagnosis and timely treatment, represents an aspect of active investigation in immunotherapy. Scientists have explored clinical parameters (gender, preexisting autoimmune disease, etc.) as well as laboratory biomarkers (absolute lymphocyte count, NLR, etc.) that are associated with increased risk of irAEs.^120,121^ However, many studies present conflicting findings. Recently, some novel biomarkers have been shown promise for clinical application. First, CD8 T cells clonal expansion in the peripheral blood could predict the development of irAEs. Subudhi *et al*. found that in prostate cancer patients treated with ICIs, expansion of ≥ 55 CD8 T cell clones preceded the development of grade 2–3 irAEs.^122^ Additionally, detection of autoantibody in the serum is another potential predictor for the occurrence of irAEs. In a study of 92 patients with NSCLC receiving the anti-PD1 mAb nivolumab,^123^ detection of more than one of autoantibodies (including ANA, ENAs and ASMA) within 30 days of starting therapy was correlated with the risk of irAEs (P = 0.002). Furthermore, several studies indicated that the baseline gut microbiome might predict immune-related colitis in patients treated with ICIs.^124,125^ Further validation studies are needed for these specific biomarkers in larger-scale cohorts of NSCLC patients receiving immunotherapy.

Exploring approaches to limit irAEs is another area of active investigation in immunotherapy. Changing dose and schedule is the most acceptable and easy to implement. For example, using lower and/or less frequent dosing of ipilimumab could maintain therapeutic benefit but reduce irAEs in the Checkmate 227 trial for NSCLC.^78^ Besides, early intervention is another feasible approach to prevent some fatal irAEs. For example, in a retrospective study of patients who developed immune-related colitis, patients receiving immunosuppression early (≤10 days) required fewer hospitalizations (P = 0.03), experienced steroid taper failure less frequently (P = 0.03), had a shorter course of steroid treatment (P = 0.09) and a shorter duration of symptoms (P < 0.01) compared with patients receiving immunosuppressive therapy > 10 days after onset of colitis.^126^ In addition to these two approaches, others including prophylactic use of drugs (such as vedolizumab), repurposed drugs (such as tofacitinib), alternative checkpoints and tumor-targeted ICIs, are also being explored to limit irAEs.^127^

### Dealing with immune resistance

With the gradual widespread clinical application of ICIs in NSCLC, immune resistance is observed in subsets of patients. Some do not respond to the inhibitors at all; for the initial responders, a substantial proportion ultimately relapse with lethal drug-resistant diseases, months or years after administration of the ICIs.

Due to the complex resistance mechanisms of immunotherapy, there is still no standardized solution to this problem. Currently, combination therapy to reverse or slow down immune resistance is the most effective measure, including combination of different types of ICIs (i.e. PD-1 inhibitor plus CTLA-4 inhibitor) or combination of ICIs with other types of therapy (i.e. chemotherapy, radiotherapy, molecular targeted therapy, anti-angiogenesis therapy). For example, combination of CTLA-4 inhibitor ipilimumab with PD-1 inhibitors nivolumab is promising as first-line treatment in advanced NSCLC.^78,128^ Due to different mechanisms of action, the combination of PD-1 and CTLA-4 inhibitor can play a synergistic effect, which can not only induce the production of a large number of T cells by antagonizing CTLA-4 at the early stage of immune response, but also restore the killing function of T cells to tumor cells by blocking the binding of PD-1 and PD-L1, and reducing T cell depletion. Exploring new therapy strategies is another important way to conquer immune resistance. T cells genetically equipped with TCRs have shown great potential in treating solid tumors including NSCLC. Therapeutic vaccines against cancer have also been explored. However, challenges including weak immunogenicity, systematic toxicity, and off-target effects remain as barriers to their clinical translation.^129^

## Conclusions

The extraordinary clinical outcomes through the application of ICI regimens make us believe that immunotherapy will constitute a more and more widely-used treatment strategy for NSCLC in the near future. The next step is to better screen potential benefit population, objectively evaluate response to immunotherapy, manage immune-related toxicity and deal with immune resistance. The exploration of new biomarkers or models to predict the efficacy, awareness of unconventional response patterns for immunotherapy, and the development of new immunotherapy including ACT therapy and cancer vaccine will improve the application of immunotherapy in clinical practice; and the ongoing trials and studies about new treatment strategies with existing and novel drugs promise to improve the precision, efficacy and safety of immunotherapy in NSCLC.
